# Persistent Priapism

**Published:** 1881-01

**Authors:** 


### PERSISTENT PRIAPISM.

A case of this was recorded in the New Orleans *Medical and Surgical Journal*, October, 1880, by Dr. A. Maguire. The patient was fifty-six years of age, strong and vigorous, without any signs of leucocythemia. The attack lasted six weeks and disappeared spontaneously. None of the many remedies used had any effect. The pain was somewhat relieved by anodynes locally. The disease seemed self-limited, and without concurrent lesions. Such cases are rare, and as yet quite inexplicable.

The two Bogus Diploma Mills of Philadelphia, the Eclectic Medical College and the American University of Medicine, are at last dead, by a decision of the Courts. Buchanan says they have issued 40,000 diplomas.

Bromide of Ethyl will produce local anesthesia, when projected on the surface with an atomizer.

